# Puromycin labeling does not allow protein synthesis to be measured in energy-starved cells

**DOI:** 10.1038/s41419-017-0056-x

**Published:** 2018-01-18

**Authors:** Ran Marciano, Gabriel Leprivier, Barak Rotblat

**Affiliations:** 1 Department of Life Sciences, Ben Gurion University of the Negev, Beer Sheva, Israel; 2 Clinic for Pediatric Oncology, Hematology and Clinical Immunology, Medical Faculty, Heinrich Heine University, Düsseldorf, Germany

To the Editor

Protein synthesis is a fundamental, tightly regulated cellular process, and several methods have been employed to measure the rate of protein synthesis in cells. One of the most well-established methods entails pulsing cells with radiolabeled amino acids, such as [^35^S]methionine and [^35^S]cysteine, for a certain amount of time, and then measuring their incorporation into newly synthesized proteins by quantifying radioactivity. The major disadvantages of this method are that it involves radioactive materials, it is not compatible with fluorescent detection, and it does not allow global identification of newly synthesized proteins by mass spectroscopy. An alternative, nonradioactive approach is based on using the synthetic methionine homolog l-azidohomoalanine (AHA), which harbors an azide group that incorporates into newly synthesized proteins. The use of click chemistry allows us to covalently label the incorporated AHA with reagents, such as biotin, for purification or detection. The amount of biotin-labeled newly synthesized proteins is determined by using tagged streptavidin and immunoblotting, fluorescence-activated cell sorting (FACS), or fluorescent microscopy^1,2^.

In 2009, Schmidt et al.^3^ published a method—termed surface sensing of translation, or SUnSET—for measuring the rate and localization of protein synthesis based on the incorporation of puromycin to newly synthesized proteins and its detection with anti-puromycin antibodies. In SUnSET, a cell culture is pulsed with puromycin during which the puromycin is incorporated into the elongating peptides; this process leads to the termination of mRNA translation, upon which the puromycin-labeled truncated peptides are released from the ribosome. The amount of puromycin-labeled peptides is then determined by using anti-puromycin antibodies and immunoblot, FACS, or fluorescent microscopy, and is presumed to reflect the rate of protein synthesis^3^. This method offers several advantages over the exogenous amino acid-based approaches, as it is inexpensive, it does not require methionine depletion prior to labeling, and it reduces the need for post-labeling sample processing. Nevertheless, while the incorporation of synthetic amino acids into elongating peptide chains does not dramatically interfere with the mRNA translation process, puromycin incorporation effectively terminates the mRNA translation elongation of the labeled peptides.

Because we are interested in investigating mRNA translation under conditions of energetic stress which have been shown to inhibit protein synthesis^4,5^, we compared puromycin labeling to AHA labeling in cells growing under various conditions, including energy starvation^4^. To this end, we used two cell types that are commonly used in cell biology research: human embryonic kidney 293 (HEK293) cells and immortalized (*p53* −/−) mouse embryonic fibroblasts (MEFs). The cells were grown in a basal medium or treated for 3 h with either glucose starvation (Dulbecco's modified Eagle's medium (DMEM) without glucose and pyruvate, supplemented with 10% dialyzed fetal bovine serum (FBS)), 2-deoxy-glucose (2DG; 25 mM), total starvation (Hanks' balanced salt solution (HBSS)-HEPES; no glucose, amino acids, or serum)^6^, or cycloheximide (CHX; 10 μg/ml). In addition, to allow for the optimal incorporation of AHA, the cells were methionine-depleted (DMEM with or without glucose and pyruvate, without methionine, and supplemented with 10% dialyzed FBS) throughout the entire 3-h treatment. The cells were then pulsed with AHA (50 μg/ml) for 130 min, and puromycin (10 μg/ml) was added for the last 10 min (Fig. 1a). The cells were lysed and AHA-incorporated proteins were labeled with biotin alkyne, using a click reaction^1^. The samples were then subjected to sodium dodecyl sulfate-polyacrylamide gel electrophoresis, and protein synthesis was detected with either streptavidin-horse radish peroxidase (to detect AHA) or anti-puromycin antibodies (Hybridoma bank clone PMY-2A4-S) (Fig. 1a).

**Fig. 1:**
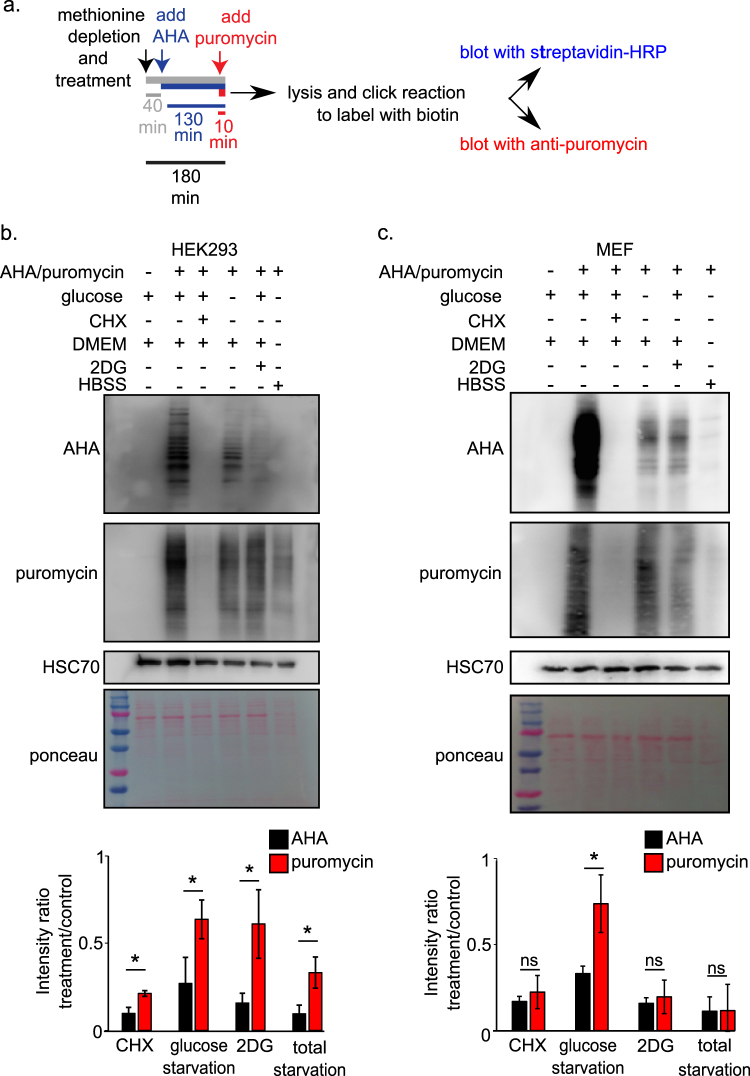
Measurement of protein synthesis rates in energy-depleted cells to compare AHA and puromycin labeling methods **a** To simultaneously measure mRNA translation under various treatments using the two methods, cells were treated and incubated with AHA and puromycin for the indicated durations. Following cell extraction, AHA-labeled proteins were tagged with biotin by using the Click-It protocol. The incorporation of both AHA and puromycin into newly synthesized proteins was detected by immunoblotting with streptavidin-HRP and with an anti-puromycin antibody, respectively. HSC-70 immunoblotting and Ponceau staining were used as loading controls. Protein synthesis rates were quantified by measuring the signal intensity in each lane using ImageJ and normalizing the values to that of the control lane. **b** Overall protein synthesis rates in HEK293 cells under the indicated treatments, as measured by AHA and puromycin labeling. Data represents mean ± SD; *p < 0.05; *n* = 3 independent experiments. **c** Overall protein synthesis rates in MEFs under the indicated treatments, as measured by AHA and puromycin labeling. Data represents mean ± SD; *p < 0.05; *n* = 3 independent experiments

Measurements of AHA labeling in HEK293 cells indicated that protein synthesis rates were dramatically reduced under all treatments compared with the basal medium (Fig. 1b). In line with the findings of Schmidt et al.^3^ and with our AHA data, puromycin labeling indicated dramatically reduced mRNA translation rates under the CHX treatment (Fig. 1b). However, to our surprise, puromycin labeling indicated only minor, significantly less dramatic reductions in protein synthesis rates (relative to those observed using AHA labeling) under glucose starvation, 2DG treatment, and total starvation conditions (Fig. 1b), none of which were tested by Schmidt et al.^3^. Similar differences were also observed between AHA- and puromycin-labeled MEF cells treated with glucose starvation: only AHA labeling indicated a substantial decrease in protein synthesis rates compared with those in the basal medium condition (Fig. 1c). Notably, puromycin labeling did indicate reduced mRNA translation in the 2DG and total starvation treatments in these cells. Finally, we tested if there are similar differences between puromycin and Click-It labeling under glucose starvation in a breast cancer cell lines, MCF7, and found that this indeed the case (Fig. S1). Together, these findings indicate that, in contrast to the high reliability of AHA labeling (Figs. 1b, c), puromycin labeling is not reliable across cell types and does not accurately measure mRNA translation rates in energetically challenged cells, particularly those under glucose starvation conditions.

Based on these data, we conclude that puromycin labeling is not suitable for measuring the rates of overall protein synthesis under conditions of energy starvation, especially glucose starvation, and that the method should be compared with exogenous amino acid incorporation experiments when employed under new experimental conditions.

## Electronic supplementary material

Supp Figure 1

Supplementary Figure Legend
