# Supplementary figures and images for: Puromycin labeling does not allow protein synthesis to be measured in energy-starved cells
Source: Cell Death Dis. 2018 Jan 18;9(2):39. doi: 10.1038/s41419-017-0056-x (PMC5833866; doi:10.1038/s41419-017-0056-x)

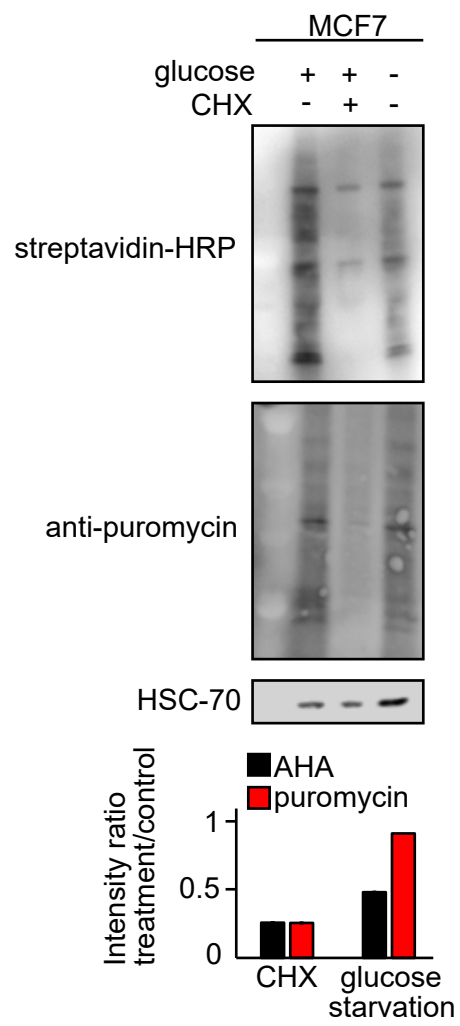

Supplement: Supplementary file 1 — Supp Figure 1 [file 41419_2017_56_MOESM1_ESM.pdf]
