# Supplementary material for: Puromycin labeling does not allow protein synthesis to be measured in energy-starved cells
Source: Cell Death Dis. 2018 Jan 18;9(2):39. doi: 10.1038/s41419-017-0056-x (PMC5833866; doi:10.1038/s41419-017-0056-x)
Supplement: Supplementary file 2 — Supplementary Figure Legend [file 41419_2017_56_MOESM2_ESM.docx]

**Supplementary Figure Legend**

**Figure S1:** **Measurement of protein synthesis rates in glucose starved breast cancer cells to compare AHA and puromycin labeling methods.** Overall protein synthesis rates in MCF7 cells under the indicated treatments, as measured by AHA and puromycin labeling as in Fig. 1a.
